# Enhancing Silicon Anode Performance in Lithium-Ion Batteries Through Hybrid Artificial SEI Layer and Prelithiation

**DOI:** 10.3390/nano15090690

**Published:** 2025-05-02

**Authors:** Bo Peng, Weizhai Bao, Kaiwen Sun, Jin Xiao

**Affiliations:** 1 School of Metallurgy and Environment, Central South University, Changsha 410083, China; bopeng@csu.edu.cn (B.P.); 130231@csu.edu.cn (J.X.); 2 School of Chemistry and Materials Science, Nanjing University of Information Science and Technology, Nanjing 210044, China; 3 Australian Centre for Advanced Photovoltaics, School of Photovoltaic and Renewable Energy Engineering, University of New South Wales, Kensington, NSW 2052, Australia

**Keywords:** prelithiation, artificial SEI layer, interfacial engineering, coulombic efficiency, silicon anode

## Abstract

Prelithiation has been widely accepted as one of the most promising strategies to compensate for the loss of active substance and to improve the initial Coulombic efficiency in silicon-based anodes for advanced high-energy-density batteries. But because of their unstable solid electrolyte interface (SEI) layer and low initial Coulombic efficiency, they expand in volume during prelithiation and react with moisture, which makes commercialization a difficult process. Herein, we have developed a strategy using lithium bis(fluorosulfonyl)imide (LiFSI) treatment to eliminate redundant lithium and generate LiF-based inorganic compounds on the surface of the prelithiated electrode. Such method not only reduces the reactiveness of the prelithiated anode but also enhances the ionic conductivity of the SEI. The rich LiF surface works as an artificial SEI, and according to electrochemical evaluation, the initial Coulombic efficiency of the prelithiated silicon anode treated with LiFSI can reach 92.9%. This technique not only increases the battery’s energy density but also its cycle stability, resulting in superior capacity retention and a longer cycling life.

## 1. Introduction

High-energy-density batteries are becoming more and more in demand as a result of the quick expansion of portable electronics and renewable energy sources. Of all the anode materials, silicon (Si) has garnered the most interest because of its superior theoretical capacity. Si has an approximate theoretical capacity of 4200 mAh/g, more than ten times that of conventional graphite anodes [1]. Although silicon anodes offer a vast amount of energy potential, there are a number of important problems that limit their use in real-world applications. Silicon has substantial volume expansion (>400%) during the lithiation and delithiation phases of the cycling process [2]. This can cause mechanical fragmentation and the loss of electrical contact, which in turn can lead to capacity deterioration. Furthermore, the electrolyte becomes unstable at the anode’s working potential, reducing to create a SEI on the anode surface. The SEI is a complex mixture of organic and inorganic lithium compounds. The issue is made worse by the unstable SEI layer that results from electrolyte breakdown [3,4]. This layer keeps consuming lithium ions while destroying the electrode–electrolyte contact. The new Si surface is vulnerable to parasitic reactions and loss of active lithium because the brittle inorganic materials, LiO and LiF, which comprise the majority of the old SEI layer, break under repetitive strain during cycling [5].

Researchers have suggested a number of optimization techniques to overcome these constraints, and prelithiation technology has proven to be a successful one [6]. Prelithiation reduces irreversible capacity loss and supplies extra active lithium to make up for lithium used during SEI rupture by adding lithium ions during the early stages of battery development. By precisely controlling lithium insertion and guaranteeing appropriate anode conditions during the first charge–discharge cycle, an ideal prelithiation technique can greatly improve energy efficiency, cycling stability, and battery longevity [7].

Multiple prelithiation techniques have been devised, such as cathode prelithiation through additives, chemical or electrochemical prelithiation pathways, and the use of lithium foil or powder for anode prelithiation [8]. Nevertheless, practical implementation of these strategies faces challenges, such as inadequate control over lithium insertion uniformity and quantity, resulting in residual lithium [9]. The recurrent SEI fracture brought on by silicon–carbon anode volume growth is not resolved by residual lithium, even if it might improve the initial active lithium supply. The SEI layer becomes more unstable with extended cycling and storage, showing increased thickening, inorganic component enrichment, and organic constituent depletion. Capacity loss and increased internal resistance are caused by these structural alterations [10,11]. The inclusion of functional additives, electrolyte solvent modification, and nanomaterial incorporation have all been used in attempts to increase SEI stability, but the results have not been ideal [12]. For instance, ether-based electrolytes (e.g., DME/DOL) can compact SEI layers and reduce the pore diameter but fail to fundamentally address these issues. In contrast, fluorinated electrolyte additives demonstrate superior efficacy in modulating SEI composition and structure, minimizing interfacial side reactions on silicon oxide anodes, and thereby improving cycling stability and rate capability. Common additives, such as fluoroethylene carbonate (FEC) and lithium bis(fluorosulfonyl)imide (LiFSI), significantly reduce SEI porosity, forming denser SEI layers [13]. Yang et al. [14] revealed that diethyl fluoromalonate, leveraging its favorable decomposition kinetics, rapidly generates a LiF-rich passivation layer at the silicon oxide interface during early SEI formation. This effectively suppresses parasitic reactions and yields an adaptive SEI layer capable of accommodating volume changes while maintaining stability over extended cycling. In addition, Wang et al. [15] proposed an innovative approach to engineer lithium bis(fluorosulfonyl)imide (LiFSI)—a material featuring fluorosulfonyl (-SO_2_F) groups and lithium imide salt structures—onto lithium metal anode surfaces via a chemical bonding strategy. The Li-F/Li-S-bonded interfacial layer, formed through in situ reactions between sulfonyl groups in LiFSI and metallic lithium, effectively suppresses lithium dendrite growth and enhances the mechanical stability of the SEI. The study further demonstrated that the LiFSI concentration critically modulates SEI layer thickness and modulus, significantly improving cell performance [16].

**Figure 1:**
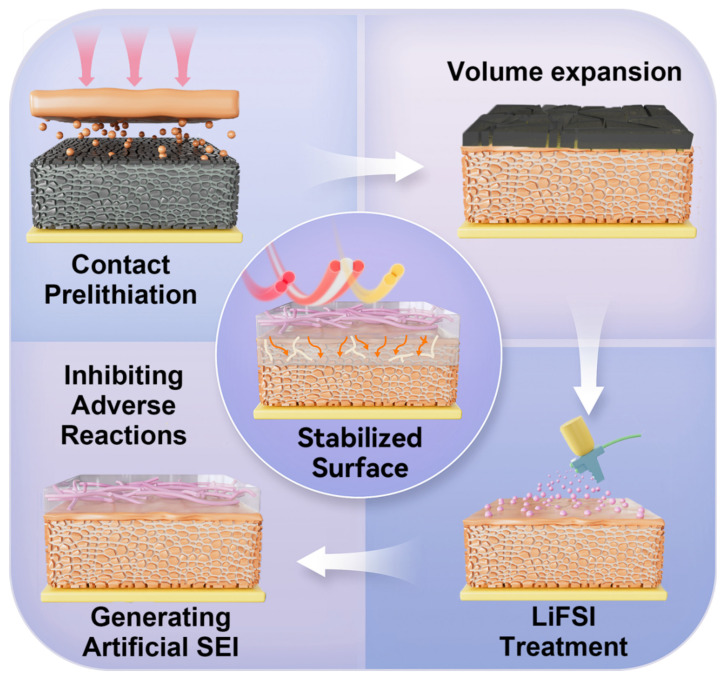
Schematic showing the LiFSI treatment process.

This study proposes an LiFSI-based prelithiation optimization strategy to address residual lithium and insufficient SEI stability in silicon–carbon anodes during cycling. A schematic diagram of the LiFSI-based modification technique is displayed in Figure 1. LiFSI and its decomposition product, fluorosulfonyl (-SO_2_F) groups, exhibit enhanced electrostatic affinity for Li^+^, effectively consuming residual lithium and accelerating SEI formation. Concurrently, LiFSI functional groups bind to silicon and graphite surfaces, forming a stable electrode network that significantly reinforces SEI mechanical integrity. The experimental findings show that the LiFSI-treated prelithiated silicon–carbon anode has a high initial Coulombic efficiency. It rose from 83.4% (the conventional prelithiation) to 92.9%. These findings not only validate LiFSI’s pivotal role in constructing high-stability SEI layers but also advance novel methodologies for anode prelithiation, offering substantial theoretical and practical implications for next-generation LIB development.

## 2. Experiment

### 2.1. Sample Preparation

To create a negative electrode slurry, we combined and stirred the conductive agent Super P carbon black (SP, 3 wt%), the binder carboxymethyl cellulose sodium (CMC, 1.5 wt%), styrene butadiene rubber (SBR, 2.5 wt%), and silicon-based active material (93 wt%). To create a negative electrode sheet, we evenly covered the slurry with 10 µm-thick copper foil and vacuum-dried it for 6 h at 100 °C. Via roller pressing, a commercial lithium strip that is 3 µm-thick was joined with the manufactured electrode sheet. To guarantee adequate lithium absorption and produce a Pre-Li sample, the composite electrode sheet was vacuum-sealed and allowed to stand at room temperature for a full day. Commercial LiFSI (purity 99.8%) was mixed with DMC at a mass ratio of 1:4, and the mixture was ultrasonicated for 10 min to achieve uniform dispersion of LiFSI in the solution, obtaining a 20 wt% LiFSI reagent. Finally, the reagent containing 20% LiFSI was evenly applied to the surface of the Pre-Li sample and kept in a vacuum oven at 80 °C for 6 h to ensure complete evaporation of the solvent, obtaining the sample.

### 2.2. Material Characterization

The prelithiated electrodes treated with LiFSI were characterized by X-ray diffraction (XRD; Bruker D8 Advance, Bruker AXS GmbH, Karlsruhe, Germany) between 10° and 90°. A scanning electron microscope (SEM; HITACHI SU8010, Hitachi High-Technologies Corporation, Tokyo, Japan) was used to examine the elemental distribution and shape of various electrode materials. We utilized X-ray photoelectron spectroscopy (XPS; Thermo Scientific K-Alpha, Thermo Fisher Scientific Inc., Waltham, MA, USA) to document the functional group distribution. The surface of the prelithiated electrode after LiFSI treatment was further characterized by TOF-SIMS (TOF-SIMS; PHI nano TOF II, ULVAC-PHI Inc., Chigasaki, Japan), giving a 50 nm-depth-profile conducted at a speed of 0.2 nm/s to obtain the distribution information of elements with depth.

The treated anode sheets were combined with lithium cobalt oxide cathode sheets (capacity: 166 mAh/g) using commercial electrolytes, and the results were tested by laminating soft-pack complete cells to confirm the samples’ effect on electrical performance. The battery’s initial efficiency data and charge–discharge voltage curves were acquired on the Neware apparatus at a rate of 0.04 C (charging cut-off voltage 4.5 V and discharging cut-off rate 0.01 C). Additionally, at 25 °C, the battery’s long-cycle capacity retention rate was acquired at a charge–discharge rate of 0.5 C. The battery’s performance was compared at 0.5 C, 1.0 C, 1.5 C, and 2.0 C (charging cut-off voltage 4.5 V and discharging cut-off rate 0.025 C). On the electrochemical workstation (CS310X-8, Corrtest Instruments Corp., Ltd., Wuhan, China), within the voltage range of 2.7–4.7 V, the cyclic voltammetry curves of the cells were obtained in the positive sweep mode at a scan rate of 0.1 mV/s.

## 3. Results and Discussion

**Figure 2:**
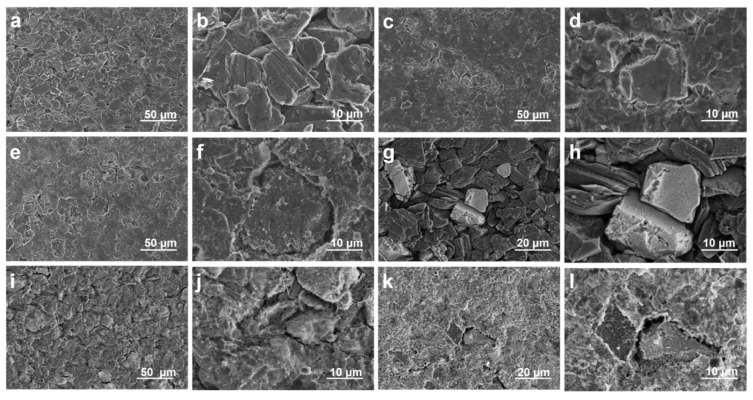
After adding lithium, the SEM displayed the original sample (**a**,**b**), the sample without surface treatment (**c**,**d**), and the sample with surface treatment (**e**,**f**). Following lithium supplementation, the backscatter SEM displayed the original (**g**,**h**) sample, the (**i**,**j**) sample without surface treatment, and the (**k**,**l**) sample with surface treatment.

To investigate the impact of LiFSI treatment on the surface structure and morphology, SEM and back SEM were used to view images of raw, prelithiated, and LiFSI-treated silicon anodes (with sizes of approximately 10 um, 20 um, and 50 um, respectively), as illustrated in Figure 2.

Due to the high solubility of LiFSI in polar electrolytes and the interfacial activity of its fluorosulfonyl groups (-SO_2_F), we proposed uniform deposition of LiFSI onto prelithiated silicon-based surfaces to ensure homogeneous contact with the anode material. The hydrophilic sulfonic acid groups (-SO_2_F) in LiFSI act as electrolyte additives, enhancing ionic conductivity and interfacial stability.

In order to verify the promoting effect of LiFSI on the formation of stable SEI film on the negative electrode surface, we tested the SEM images of the negative electrode surface under full charge after two and a half cycles of charging and discharging at a small rate of 0.04 C. It can be found that SEI film was almost invisible on the surface of the original silicon–carbon-negative electrode, and the expanded silicon–carbon particles caused large-scale cracks on the surface (Figure 2a,b). By EDS testing, it was found that the original electrode surface contained less of the F element required to construct the SEI film (Figure S1). However, the coverage of SEI film on the surface of the pre-lithium silicon–carbon anode only increased (Figure 2c,d). EDS tests showed a slight increase in the fluorine intensity on the electrode surface (Figure S2). This is due to the fact that pre-lithium can provide more active lithium, which can accelerate the formation of the SEI film [17]. However, it did not change the structure of the SEI film, and the mechanical strength of the SEI film was not improved. Therefore, the expanded silicon–carbon particles made the negative electrode surface show large-scale cracks. At the same time, we noticed that the intensity of the S element on the surface of the pre-lithium electrode increased significantly. This is because the surface of the electrode is residual by pre-lithium, and in the preparation of the SEM test stage, the electrode will inevitably contact the air, resulting in oxidation [18]. The surface of the pre-lithium +6 M LiFSI electrode saw obvious SEI film covering the electrode surface, and the surface cracks caused by volume expansion were effectively covered (Figure 2e,f). EDS images also showed that the F element on the electrode surface was significantly increased (Figure S3). This suggests that LiFSI promoted the formation of SEI films with high stability and ionic conductivity. That is, (1) LiFSI will decompose preferentially and participate in the construction of inorganic-rich SEI components. (2) The sulfonimide groups of LiFSI will coordinate with Li^+^ ions to form a stable ionic transport network. At the same time, the decomposition products of LiFSI also exhibit self-healing properties, which can mitigate the SEI film rupture caused by volume expansion.

**Figure 3:**
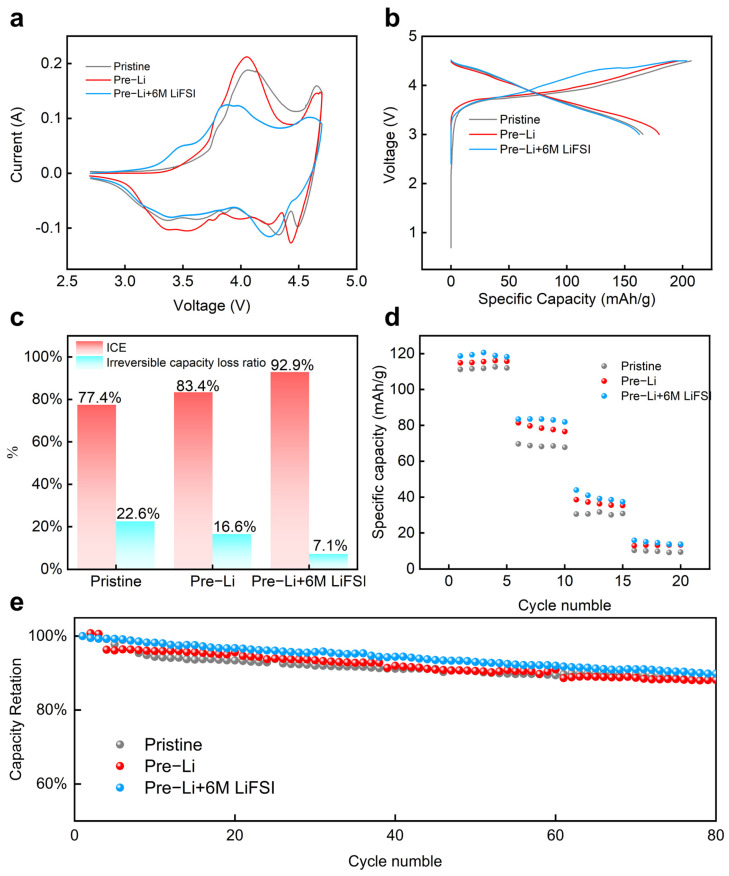
The electrochemical performance of variously treated samples. (**a**) CV, (**b**) voltage profile, (**c**) Coulombic efficiency and irreversible capacity, and (**d**) rate capability from 0.5 C to 2 C. (**e**) Cycling performance of different electrodes.

The surface alterations brought about by the LiFSI treatment were further supported by backscattered SEM images of the same samples, which are shown in Figure 3. The untreated prelithiated anode’s backscattered SEM images (Figure 2i,j) displayed contrast changes, pointing to regions of varying composition or oxidation states that were most likely the consequence of side reactions and moisture exposure. The LiFSI treatment anode, on the other hand, had a more uniform structure with less compositional variation (Figure 2k,l), indicating that the passivating layer was successfully formed. Reducing parasitic reactions and preserving the stability of the electrode–electrolyte contact depends on this homogeneity.

It is evident from the comparison of SEM and backscattered SEM pictures that the artificial SEI layer, which was created by the LiFSI spraying method, greatly enhanced the integrity and surface morphology of the prelithiated silicon anode. The LiFSI treatment is essential for increasing the overall electrochemical performance, especially for cycle stability and early Coulombic efficiency, because it efficiently suppresses side reactions and stabilizes the electrode surface.

Electrochemical performance was examined using single-layer LCO||Si-C/Gr pouch cells (format: 80 mm × 64.2 mm × 0.85 mm, 0.38 Ah). The cyclic voltammetry curves in Figure 3a exhibited distinct differences in peak shapes and positions between prelithiated electrodes and LiFSI-passivated prelithiated electrodes, indicating that these treatments altered the surface characteristics and internal structure of electrode materials to influence lithium-ion intercalation and deintercalation processes. The prelithiated electrode demonstrated enhanced redox peak intensities attributed to the residual lithium metal enabling more lithium ions to participate in reversible reactions, while its broader current response range reflected elevated electrochemical activity linked to accelerated SEI film formation that improved the lithium-ion transport efficiency. LiFSI-treated electrodes exhibited significantly reduced anode peak intensity at 4 eV and disappearance of parasitic peaks at 4.45 eV, resulting from sulfonyl fluoride groups consuming residual lithium while rapidly forming protective SEI layers to minimize electrode–electrolyte contact and suppress side reactions. The narrowed current response range in LiFSI-passivated electrodes implied enriched inorganic components, like LiF and Li_2_S, in SEI layers that optimizd lithium-ion transport paths and reaction kinetics [19]. Figure 3b demonstrates flatter voltage plateaus in prelithiated electrodes, indicating reduced voltage polarization beneficial for energy density enhancement, whereas the LiFSI-passivated counterparts displayed complex voltage variations in high-capacity regions with lower polarization and higher energy efficiency, corroborating the SEI modification effects observed in CV curves. The initial Coulombic efficiency increased from 77.4% for pristine anodes to 83.4% for prelithiated and 92.9% for LiFSI-passivated electrodes, with irreversible capacity losses decreasing from 22.6% to 16.6% and 7.1%, respectively, confirming LiFSI’s optimization of SEI formation stability (Figure 3c) [20]. Rate capability tests (Figure 3d) showed LiFSI-treated electrodes delivering superior capacities of 118.7, 83.5, 43.9, and 15.9 mAh g^−1^ at 0.5 C to 2 C rates due to enhanced SEI ionic conductivity and structural integrity [21]. After 80 cycles (Figure 3e), LiFSI-passivated electrodes maintained 89.81% capacity retention, outperforming pristine and prelithiated samples at 88.11% and 88.25%, with prelithiated electrodes initially exceeding 100% capacity retention from residual lithium supplementation but gradually degrading due to SEI fracture–regeneration cycles caused by electrode expansion, leading to lithium loss and impedance from thickened SEI layers [22]. These findings collectively validated that LiFSI passivation reinforced SEI stability and lithium compensation mechanisms, establishing high-performance silicon-based anodes with optimized interface dynamics.

**Figure 4:**
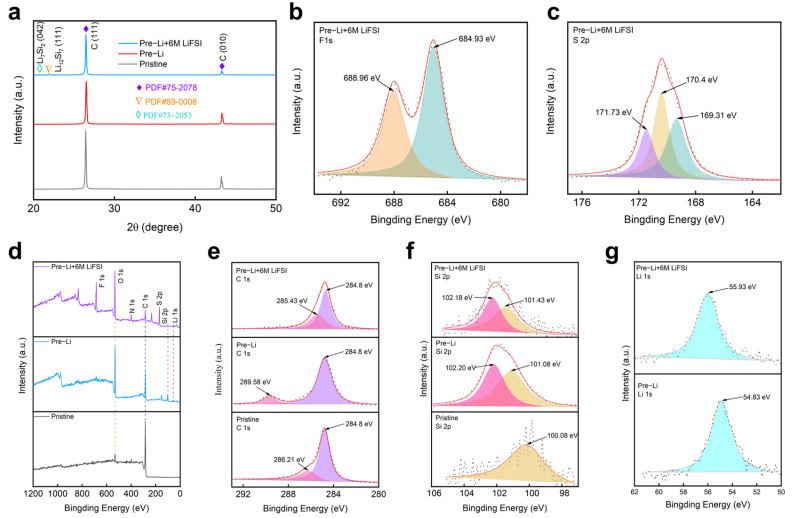
(**a**) XRD patterns of pristine material, prelithiated samples with/without surface treatment, (**b**) F1s XPS spectrum of LiFSI treatment after lithium compensation, (**c**) S2p XPS spectrum of LiFSI-coated sample after lithium compensation, (**d**) XPS survey spectra of three samples, (**e**) C1s spectra, (**f**) Si2p spectra, and (**g**) Li1s spectra comparison between surface-treated and untreated samples after lithium compensation.

In order to explore the structural changes of the material and further demonstrate the role of LiFSI, phase analysis was performed on different electrodes. The XRD pattern in Figure 4a reveals the phase evolution of the electrodes before and after prelithiation with surface treatment. The intensity of peak C significantly decreased after prelithiation, indicating structural modifications. The emergence of new lithium–silicon phases further confirmed successful lithiation [23]. Additionally, the further reduction in peak C intensity after LiFSI treatment suggested increased amorphization, which helped mitigate volume expansion and improve electrode stability. The mechanism of SEI film formation accelerated by LiFSI was further revealed by XPS experiments. As shown in Figure 4d, it can be found from the full spectrum of XRD that only the pre-lithium electrode had a weak F peak, while the pre-lithium +6M LiFSI electrode had an obvious F peak, indicating the promoting effect of LiFSI on the formation of SEI film. The F1s XPS spectrum in Figure 4b reveals the chemical composition of the SEI layer after prelithiation +6M LiFSI surface treatment. The XPS spectrum of F1s confirmed the existence of LiF after LiFSI treatment, with a peak at 684.93 eV of LiF and a peak at higher binding energies of 688.96 eV, possibly coming from residual LiFSI after surface treatment [24]. Figure 4c displays the S2p XPS spectrum of a prelithiated and LiFSI-treated Si-based anode, revealing three characteristic binding energy peaks at 171.73 eV, 170.4 eV, and 169.31 eV, corresponding to distinct sulfur chemical states.

**Figure 5:**
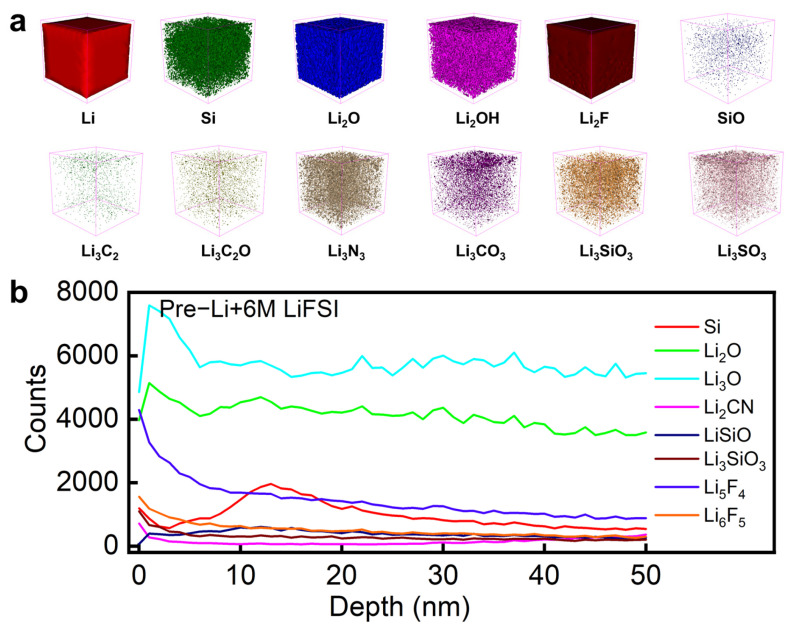
(**a**) Positive ions: 3D image and (**b**) depth profile of the prelithiated electrode after surface treatment, showing distribution of surface species.

The high-energy peak at 171.73 eV was attributed to S^6+^ species (e.g., sulfonate groups, -SO_3_^−^, or sulfate, SO_4_^2−^), indicating oxidative decomposition of LiFSI during interfacial reactions. The dominant peak at 170.4 eV corresponded to S^4+^ in the sulfonyl group (-SO_2_F) of intact LiFSI residues, which enhanced the ionic conductivity of the SEI layer. The low-energy peak at 169.31 eV originated from reduced sulfur species (e.g., Li_2_S_x_ or S^2−^), formed through lithium-mediated reduction. This multivalent sulfur evolution demonstrated LiFSI-derived gradient SEI formation, balancing ionic transport and mechanical stress buffering [25]. Figure 4e shows the XPS C1s spectrum of the three types of electrodes. Carbon peaks at 284.8 eV and 286.21 eV appeared in the spectrum of the pristine electrode, suggesting that carbon mainly formed in the C-C bonds, and carbonyl species [15,16]. The carbonyl species converted to carboxylic species (289.58 eV) after prelithiation. Additionally, the peak of carboxylic species shifted to a lower binding energy of 288.8 eV after surface stabilization by LiFSI, suggesting that the carbon species was reduced in chemical reaction with LiFSI [26]. Figure 4f shows the Si2p spectrum of the three types of electrodes. Apparently, there was SiO_x_ or LiSiO_x_ species formed after prelithiation, showing Si2p peaks at 102.2 eV, indicating high reactivity of the surface of the prelithiated electrode and its reaction with atmosphere. The peaks of SiO_x_ or LiSiO_x_ shifted to a lower binding energy of 102.18 eV after surface stabilization, suggesting reduced surface reactivity after LiFSI treatment [27]. Finally, in Figure 4g, the Li1s spectrum shows two peaks at 55.93 eV and 54.83 eV, associated with lithium compounds such as Li_x_SO_y_F_z_ and LiF. The upper spectrum represents the prelithiated electrode with LiFSI treatment, while the lower spectrum corresponds to the prelithiated electrode without surface treatment. The negligible shift and similar intensity between the two spectra suggested that LiFSI treatment did not significantly alter the total lithium content in the SEI but instead influenced its composition and distribution. This implies that the primary role of LiFSI was to optimize the SEI structure, leading to improved passivation, reduced side reactions, and enhanced cycling stability [28]. The SEI of the surface treatment electrode was analyzed using TOF-SIMS. Figure 5b shows the depth profile of the electrode in the first 50 nm, revealing SEI characteristics. It was seen that LiF located at the very first 10 nm, constituting an important component of the SEI of the prelithiated anode after surface treatment by LiFSI. Additionally, there was Li_2_CN species whose amount increased with the increasing depth, possibly coming from the reaction between the prelithiated electrode and LiFSI. Moreover, Li_3_SiO_3_ existed as a main substance in the first 50 nm SEI, likely from the prelithiation reaction and the following oxidation, consistent with the results of XPS. Finally, it was seen that Li_2_O existed as an important component in the SEI of the LiFSI-treated electrode. Figure 5a presents the SEI compositions in the format of 3D cubes. It is clearly seen that LiF aggregated at the surface of the LiFSI-treated prelithiated electrode. In addition, Li_3_SiO_3_ distributed homogeneously, likely following the curvature of silicon–carbon composites. According to the negative-ion TOF-SIMS image shown in Figure S9, sulfonyl fluoride groups formed a high concentration of LiSO_3_ on the electrode surface, which verified the XPS characterization results. In summary, the SEI layer formed by LiFSI passivation treatment was characterized by inorganic compounds, such as LiF, Li_2_O, LiSO_3_, and LiSiO_3_, thereby promoting the formation of a highly stable SEI film.

## 4. Discussion

It was revealed that prelithiation effectively enhanced the initial Coulombic efficiency and reaction kinetics. Moreover, reversible capacities, rate capabilities, and cycling stability improved when the interface of the prelithiated electrode was stabilized by LiFSI-based modifications. LiFSI treatment reacted with redundant lithium of the prelithiated electrode, contributing to a modified SEI, which was rich in LiF variants and effectively suppressed silicon’s volume expansion during cycling. Such artificial SEI had an organic core and an inorganic shell, which restricted the formation of insulating products during the prelithiation process while accommodating structural stress from silicon expansion, making the prelithiated electrode much more tolerant to humidity [29]. Also, it significantly enhanced the practical feasibility of employing contact prelithiation technologies.

Without surface treatment, the reactive prelithiated electrode would be oxidized quickly. The insulating oxidation products would passivate the electrode surface, resulting in a broken ion/electron transport pathway. For the electrode with LiFSI-induced surface stabilization, electron and ion transport pathways were retained, which amplified kinetics and maintained structural integrity against volume changes. LiFSI treatment further ensured the reaction stability of the Si-anode, limited irreversible reactions, mitigated particle pulverization caused by repeated expansion/contraction, and avoided inhomogenization during the reaction. However, it is noted that the protection effect became weaker in a harsh environment, possibly due to the fact that the established SEI was not strong enough. To guarantee long-term cycling stability against severe volume fluctuations, more effective measures are needed to tune the uniformity of surface treatment, parameters of the treatment, like temperature and time, the pretreatment process of the prelithiated electrode before contacting and reacting with LiFSI, and the post-treatment of the LiFSI-modified electrode before contacting with electrolytes.

## 5. Conclusions

In conclusion, we have developed a facile method to stabilize the reactive surface of the prelithiated Si-based anode by LiFSI. The LiFSI reacted with redundant lithium on the Si/C-Gr electrode and converted it into LiF-based species that protected the surface of the prelithiated anode. The LiF-based passivation species worked as an artificial SEI and avoided the volume expansion of prelithiated electrodes. It also potentially restricted the further extensive reaction between the prelithiated electrode and electrolyte, modifying the SEI of the prelithiated Si-based anode. The prelithiated silicon negative electrode treated with ASEI demonstrated a Coulombic efficiency (ICE) of up to 92.9% in the first cycle, which was much greater than the untreated sample, according to electrochemical measurements. Additionally, enhanced capacity retention and rate capabilities were identified, suggesting that kinetics was improved, possibly due to the retained electron/ion transport pathways. This further implied more uniform redox reactions because of the less-insulating components that existed in the initial SEI structure. We believe this facile method is of great significance to the realization of the contact prelithiated Si-anode and high-energy density batteries. More importantly, it reduces the manufacturing cost of the contact prelithiated Si-anode and contributes to a feasible technology in commercialization.

## Data Availability

The data presented in this study are available on request from the corresponding author.

## Supplementary Materials

The following supporting information can be downloaded at: https://www.mdpi.com/article/10.3390/nano15090690/s1, Figure S1. EDS images of different elements tested at full charge after two and a half cycles of the original sample at a small rate of 0.04C. (a) full spectrum, (b) carbon, (c) silicon, (d) oxygen, (e) nitrogen, (f) fluorine; Figure S2. EDS images of different elements tested in a fully charged state after two and a half cycles at a small rate of 0.04C for a sample without surface treatment after lithium supplementation. (a) full spectrum, (b) carbon, (c) silicon, (d) oxygen, (e) nitrogen, (f) fluorine; Figure S3. EDS images of different elements tested in a fully charged state after two and a half cycles at a small rate of 0.04C for surface treated samples after lithium supplementation. (a) full spectrum, (b) carbon, (c) silicon, (d) oxygen, (e) nitrogen, (f) fluorine; Figure S4. TOF-SIMS Spectra-Pre-Li+6M LiFSI; Figure S5. TOF-SIMS Positive Ions Mapping-C3; Figure S6. TOF-SIMS Positive Ions Profiles-C3; Figure S7. TOF-SIMS Negative Ions Mapping-C3; Figure S8. TOF-SIMS Negative Ions Profiles-C3; Figure S9. TOF-SIMS Negative Ions 3D Images-C3; Negative ions depth profile of the prelithiated electrode after surface treatment showing distribution of surface species; Figure S10. Negative ions depth profile of the prelithiated electrode after surface treatment showing distribution of surface species.
