# Supplementary material for: Enhancing Silicon Anode Performance in Lithium-Ion Batteries Through Hybrid Artificial SEI Layer and Prelithiation
Source: Nanomaterials (Basel). 2025 May 2;15(9):690. doi: 10.3390/nano15090690 (PMC12073230; doi:10.3390/nano15090690)
Supplement: Supplementary file 1 [file nanomaterials-15-00690-s001.zip › nanomaterials-3575506-supplementary.pdf]

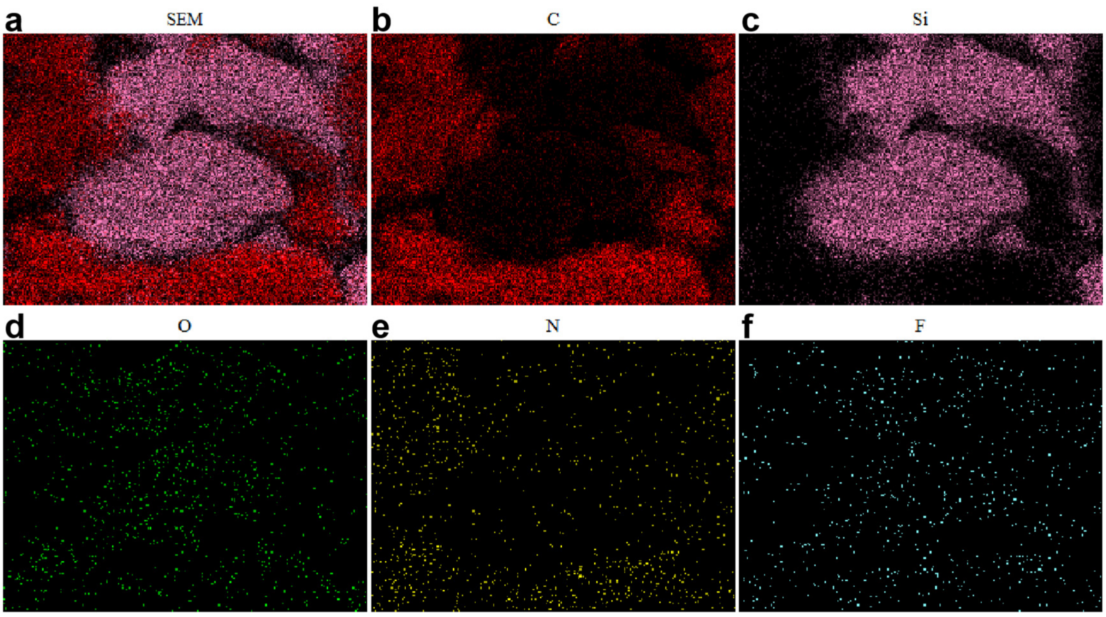


**Figure S1.** EDS images of different elements tested at full charge after two and a half cycles of the original sample at a small rate of 0.04C. (a) full spectrum, (b) carbon, (c) silicon, (d) oxygen, (e) nitrogen, (f) fluorine.

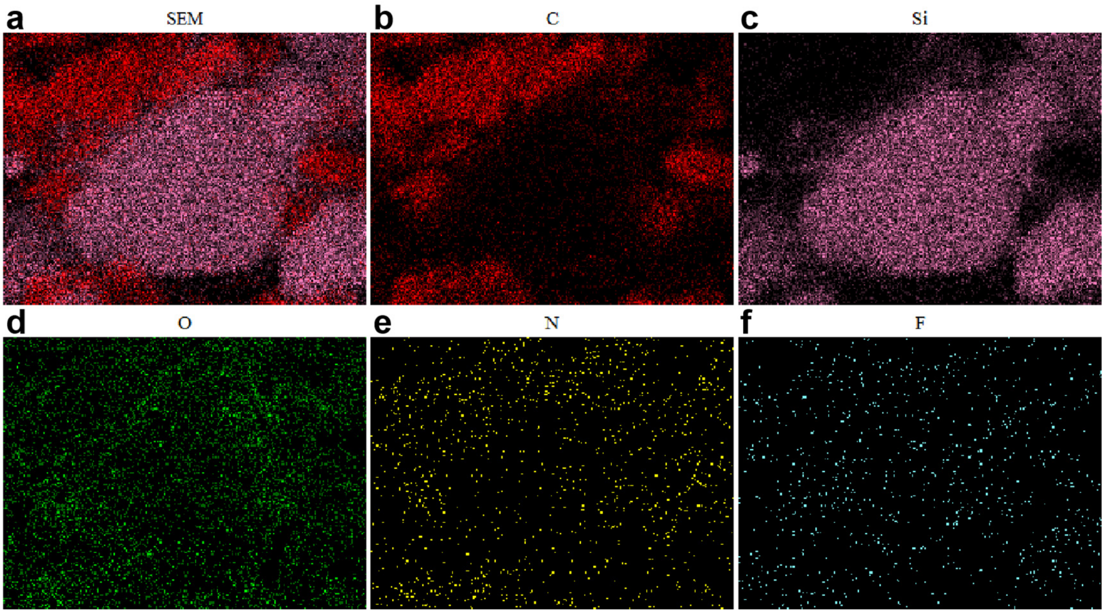


**Figure S2.** EDS images of different elements tested in a fully charged state after two and a half cycles at a small rate of 0.04C for a sample without surface treatment after lithium supplementation. (a) full spectrum, (b) carbon, (c) silicon, (d) oxygen, (e) nitrogen, (f) fluorine.

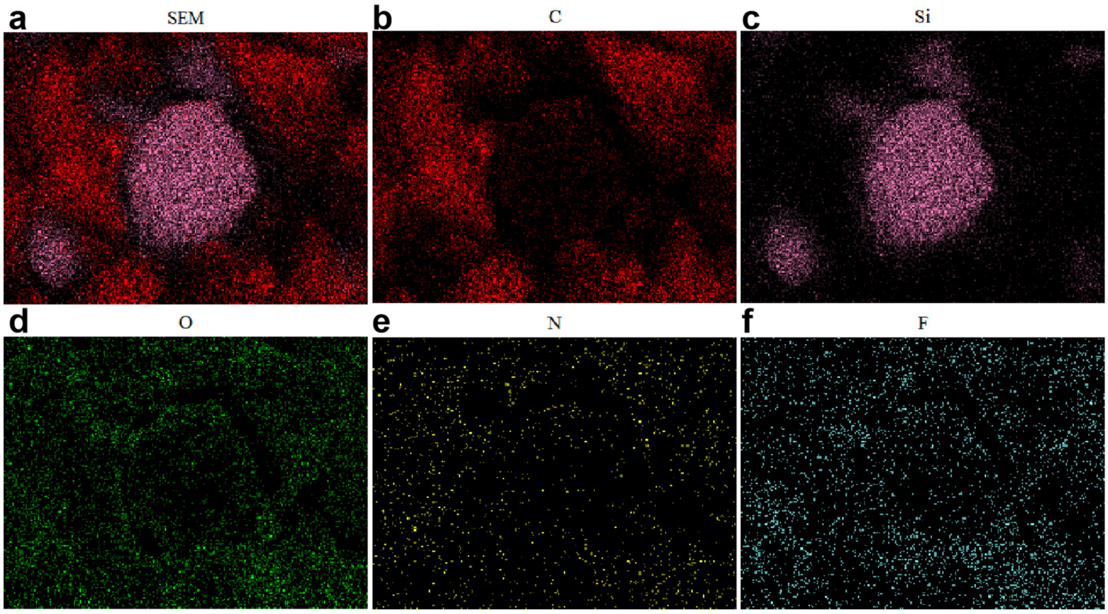


**Figure S3.** EDS images of different elements tested in a fully charged state after two and a half cycles at a small rate of 0.04C for surface treated samples after lithium supplementation. (a) full spectrum, (b) carbon, (c) silicon, (d) oxygen, (e) nitrogen, (f) fluorine.

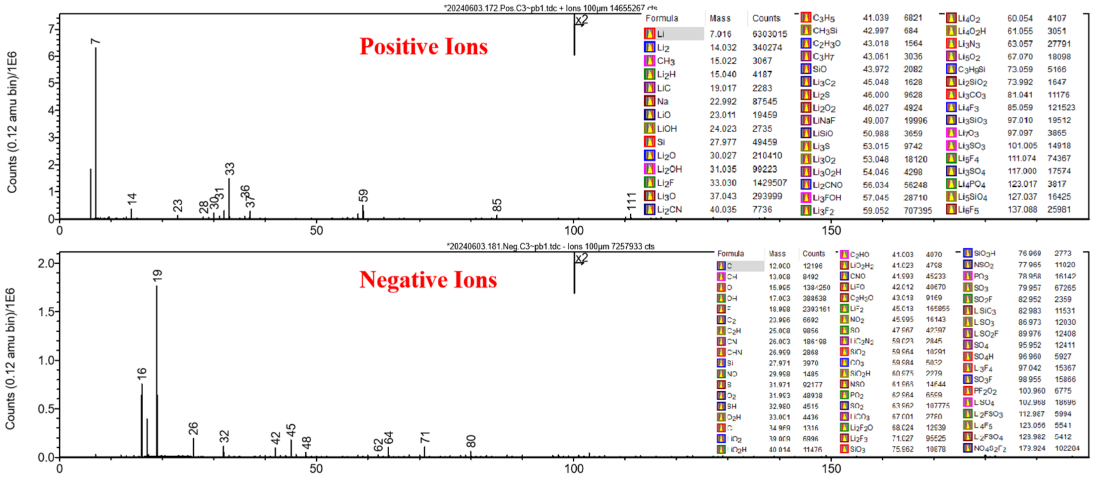


**Figure S4.** TOF-SIMS Spectra-Pre-Li+6M LiFSI

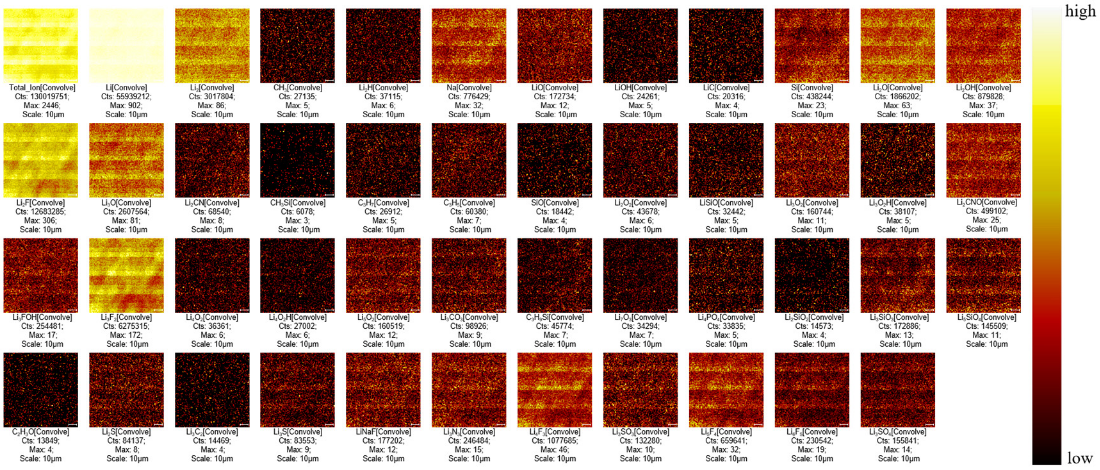


**Figure S5.** TOF-SIMS Positive Ions Mapping-C3

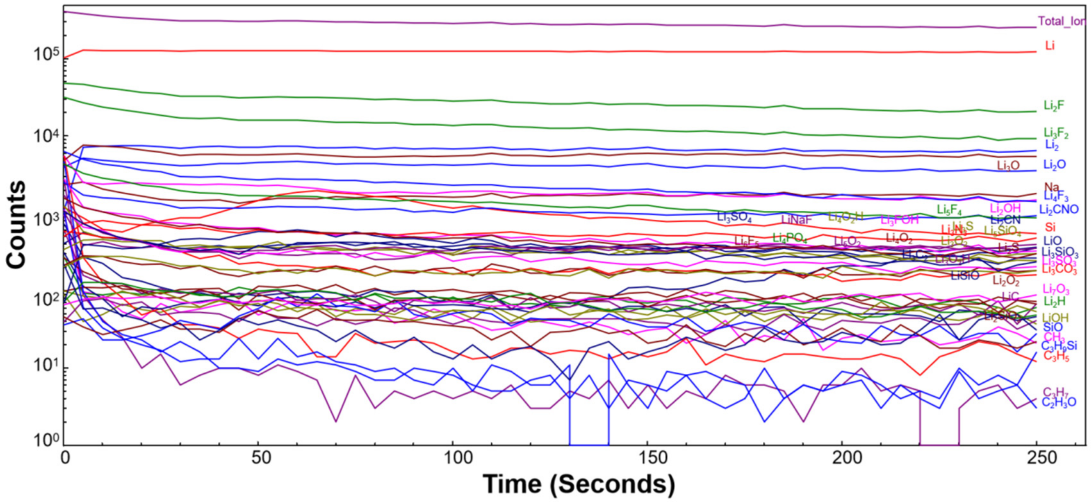


**Figure S6.** TOF-SIMS Positive Ions Profiles-C3

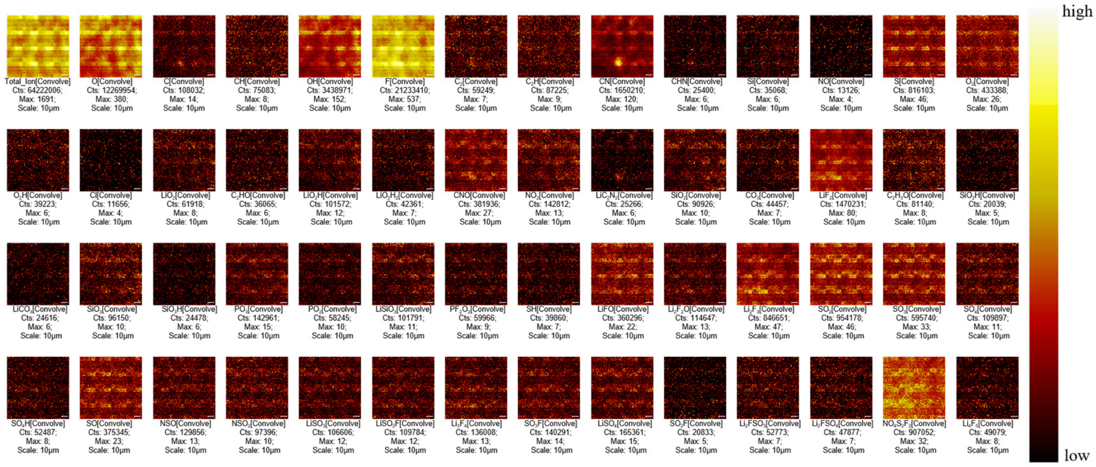


**Figure S7.** TOF-SIMS Negative Ions Mapping-C3

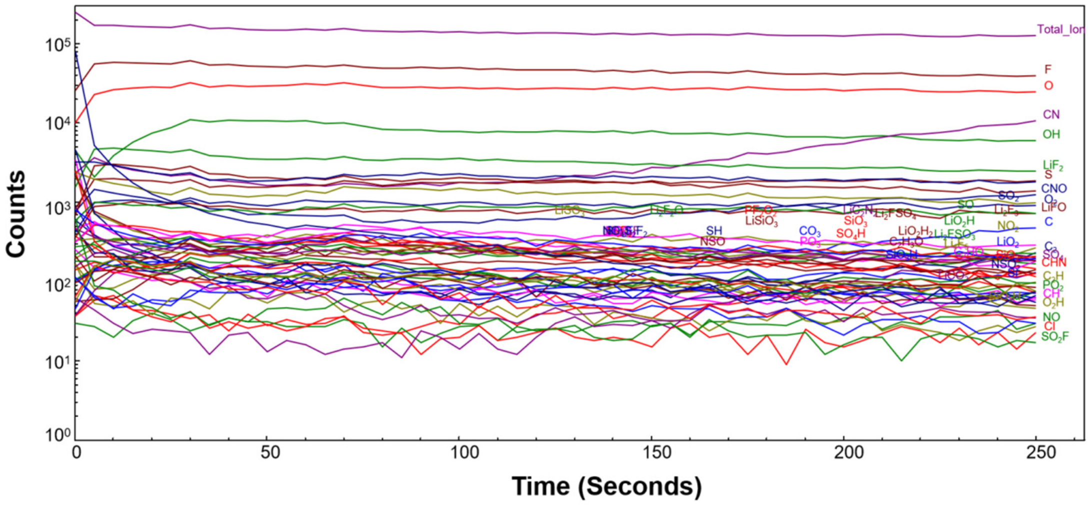


**Figure S8.** TOF-SIMS Negative Ions Profiles-C3

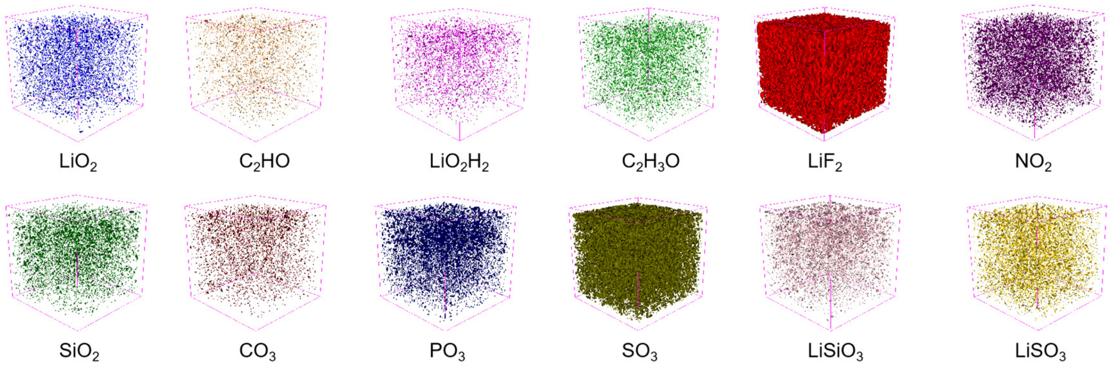


**Figure S9.** TOF-SIMS Negative Ions 3D Images-C3

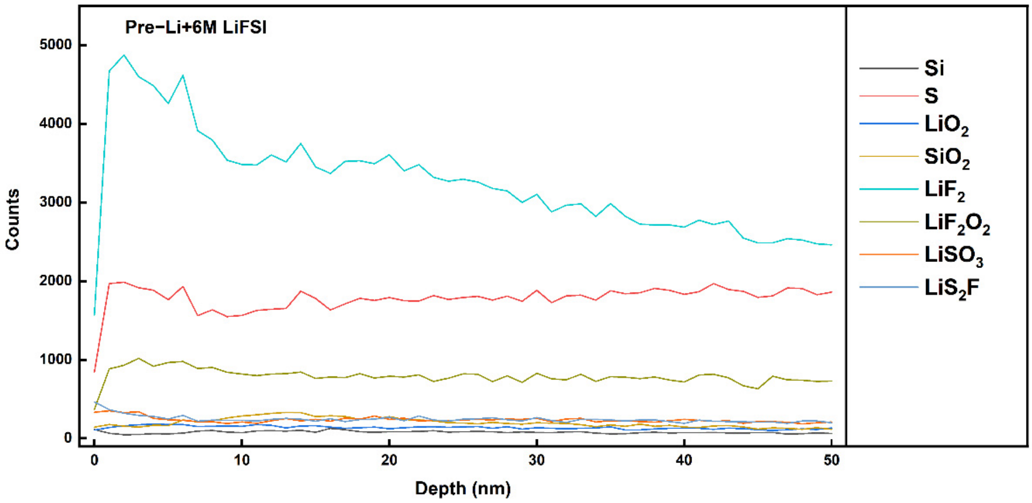


**Figure S10.** Negative ions depth profile of the prelithiated electrode after surface treatment showing distribution of surface species.
